# Versorgungsstruktur der ambulanten Urologie in Deutschland

**DOI:** 10.1007/s00120-023-02048-x

**Published:** 2023-03-09

**Authors:** Helmut Haas, Laura Müller, Thomas Speck, Maurice Stephan Michel, Johannes Huber

**Affiliations:** 1 Urologische Stiftung Gesundheit gGmbH, Düsseldorf, Deutschland; 2 Klinik für Urologie, Philipps-Universität Marburg, 35043 Marburg, Deutschland; 3 Urologische Praxis, Berlin, Deutschland; 4 Klinik für Urologie und Urochirurgie, Universitätsmedizin Mannheim, Universität Heidelberg, Mannheim, Deutschland

**Keywords:** Niederlassung, Versorgung, Urologinnen, Generationenwandel, Versorgungsforschung, Establishment, Patient care, Female urologist, Generational change, Health services research

## Abstract

**Hintergrund:**

Obwohl urologische Facharztpraxen zentraler Pfeiler der ambulanten Patientenversorgung in Deutschland sind, fehlen hierzu aktuelle Daten. Eine Beschreibung der Strukturen in Großstädten gegenüber dem ländlichen Raum sowie von Gendereffekten und Generationsunterschieden ist auch als Ausgangsbasis für weitere Untersuchungen erforderlich.

**Material und Methoden:**

Die Erhebung umfasst Daten des Arztverzeichnisses der Stiftung Gesundheit sowie der Bundesärztekammer und des Statistischen Bundesamtes. Die erfassten Praxen bzw. Kolleg*innen wurden in Subgruppen eingeteilt. Anhand der unterschiedlichen Subgruppengröße lassen sich Aussagen über die Versorgungsstruktur der ambulanten Urologie in Deutschland treffen.

**Ergebnisse:**

Während in größeren Städten die Mehrheit der niedergelassenen Urolog*innen in Berufsausübungsgemeinschaften (BAG) arbeitet und durchschnittlich weniger Patient*innen betreut, zeigt sich im ländlichen Raum ein besonders hoher Anteil an Einzelpraxen mit mehr zu versorgenden Einwohnern pro Urolog*in. Urologinnen arbeiten häufiger in der stationären Versorgung. Wenn sich Fachärztinnen für Urologie für die Niederlassung entscheiden, dann eher in BAG und eher in städtischen Gebieten. Außerdem zeigt sich eine Verschiebung der Geschlechterverteilung: Je jünger die betrachtete Alterssubgruppe, desto höher liegt der Anteil an Urologinnen unter allen Kolleg*innen.

**Schlussfolgerung:**

Wir beschreiben erstmalig die tatsächliche Versorgungsstruktur der ambulanten Urologie in Deutschland. Hierbei deuten sich Trends an, die die Art zu arbeiten und die Patientenversorgung in einigen Jahren maßgeblich beeinflussen werden.

**Zusatzmaterial online:**

Die Online-Version dieses Beitrags (10.1007/s00120-023-02048-x) enthält ergänzend zu den Analysen der Hauptarbeit die zugrunde liegenden Datenwerke und weitere Aspekte der Praxisurologie einschließlich des zeitgeschichtlichen Kontexts und der daraus abgeleiteten Trends. Abschließend erfolgt eine Analyse des Belegarztwesens.

## Abstract (en)

**Background:**

Although urological specialist practices are central pillars of outpatient care, there is a lack of current data on the care structure of these practices. A description of the structures in large cities versus rural areas as well as gender effects and generational differences is needed not only as a baseline measure for further studies.

**Materials and methods:**

The survey includes data from the physician directory of the Stiftung Gesundheit as well as from the German Medical Association and the Federal Statistical Office. Colleagues were divided into subgroups. Based on the different subgroup sizes, statements about the care structure of outpatient urology in Germany can be made.

**Results:**

While the majority of urologists in larger cities work in professional practice groups and care for fewer patients on average, in rural areas there is a particularly high proportion of individual practices with more inhabitants to be cared for per urologist. Female urologists work more frequently in the context of inpatient care. When female urology specialists choose to establish themselves, they are more likely to do so in practice groups and in urban areas. In addition, there is a shift in gender distribution: the younger the age subgroup considered, the higher the proportion of female urologists among all colleagues.

**Conclusions:**

This study is the first to describe the current structure of outpatient urology care in Germany. Future trends are already emerging that will significantly influence our way of working and the care of patients in the coming years.

## Hintergrund und Fragestellung

In Deutschland werden ambulante Patienten überwiegend in Praxen selbständiger niedergelassener Ärzte versorgt. Auch in der Urologie sind die urologischen Facharztpraxen zentraler Pfeiler der ambulanten Patientenversorgung. Regelmäßige Publikationen der Bundesärztekammer und der Kassenärztlichen Bundesvereinigung beschreiben die Rahmenbedingungen dieser Versorgung, ermöglichen allerdings nur wenig Rückschluss auf die tatsächliche Versorgungsstruktur der einzelnen Fachbereiche. Für viele praktische Fragestellungen bildet diese Versorgungsstruktur jedoch einen wesentlichen Bezugspunkt.

Um diese Ausgangsbasis für folgende Erhebungen und Analysen zu schaffen, bedarf es einer umfassenden Beschreibung der aktuellen ambulanten urologischen Versorgungsstruktur in Deutschland. Besondere Relevanz haben hierbei die unterschiedlichen Versorgungsstrukturen in Großstädten gegenüber dem ländlichen Raum sowie die sich wandelnden Bedürfnisse der Urolog*innen. Letzteres berücksichtigt die zunehmende Bedeutung von Urologinnen (Gendereffekte) und mögliche gesellschaftliche Veränderungen mit veränderten Prioritäten (Generationsunterschiede).

Ziel der vorliegenden Arbeit ist es, die Versorgungsstruktur der ambulanten Urologie in Deutschland zu beschreiben.

## Material und Methoden

Als primäre Datenquelle für die Auswertung der Versorgungsstruktur der ambulanten Urologie in Deutschland verwendeten wir das Arztverzeichnis der Stiftung Gesundheit, welche über das AOK-Arztsuche-Portal einsehbar ist [1]. Die Datenerhebung erfolgte im Februar und März 2022 durch Helmut Haas. Ergänzend analysierten wir die Statistiken der Bundesärztekammer (Stichtag 31.12.2020; [2]) und erweiterten diese Datensammlung um Informationen der regional zuständigen AOK-Niederlassungen sowie des Statistischen Bundesamtes [3, 4].

Für die Auswertung des Belegarztwesens (s. Online-Supplement) wurden aus den Analysedaten des Reimbursement Institute [5] und dem Portal urologen.net [6] Kliniken als mögliche Belegarztstandorte selektiert. In einer Internetrecherche im August 2022 wurden die Internetauftritte dieser Kliniken auf Belegärzt*innen überprüft. Zur Abgrenzung von Honorarärzt*innen und angestellten Teilzeitärzt*innen im Krankenhaus wurden nur solche Arztnamen akzeptiert, die eindeutig als Belegärzt*innen zu erkennen waren.

Da es sich in der vorliegenden Arbeit im sprachlichen Gebrauch ausschließlich um niedergelassene Fachärzt*innen für Urologie handelt, verwendeten wir hierfür im Folgenden die Begriffe „Urologin“ oder „Urologe“ bzw. in der Kurzform „FÄ-Uro“. Kolleg*innen in Weiterbildung oder in der stationären Versorgung wurden durch einen entsprechenden Zusatz beschrieben. In Abgrenzung zur Einzelpraxis (EP) beschreibt der Begriff Berufsausübungsgemeinschaft (BAG) die Zusammenarbeit von zwei oder mehr Ärzt*innen in einer Praxis. Dies schließt medizinische Versorgungszentren, Einrichtungen nach § 402 Abs. 2 SGB V (ehemals § 311 SGB V), KV-Eigeneinrichtungen und kommunale Eigeneinrichtungen mit ein. Angelehnt an die offizielle Terminologie des Bundesamtes für Bauwesen und Raumordnung [7] definierten wir „ländliche Regionen“ (< 50.000 Einwohner), „Mittelstädte“ (50.000–100.000 Einwohner), „Großstädte“ (100.000–1 Mio. Einwohner) und „Millionenstädte“ (> 1 Mio. Einwohner).

Von den veröffentlichenden Stellen erhielten wir die Erlaubnis, die erhobenen Daten wissenschaftlich auszuwerten und anonymisiert zu publizieren. Statistische Analysen erfolgten mittels R (Version 3.4.3; R Foundation for Statistical Computing, Wien, Österreich). Zum Gruppenvergleich verwendeten wir den χ^2^-Test und definierten das Signifikanzniveau mit *p* < 0,05.

## Ergebnisse

### Urolog*innen im ambulanten und stationären Bereich

**Figure 1:**
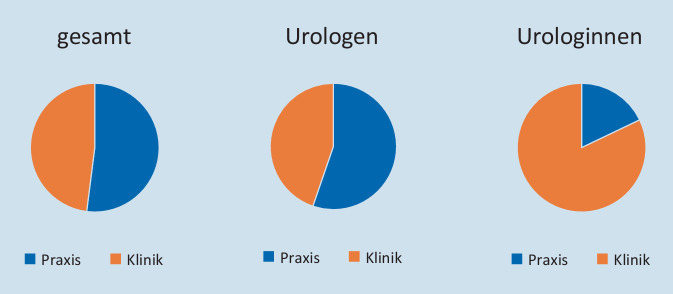

Ende 2020 waren im ambulanten und stationären Bereich 6347 Urolog*innen in der Patientenversorgung tätig. Davon waren 1270 Frauen (20 %). Abb. 1 veranschaulicht das Arbeitsumfeld der Kolleg*innen sowie die geschlechtsspezifische Verteilung. Bezogen auf alle Urolog*innen waren 52 % im ambulanten Sektor beschäftigt. Frauen wählten mit 61 % vs. 44 % der männlichen Kollegen deutlich häufiger den stationären Sektor als Arbeitsumfeld (*p* < 0,001).

### Ambulante Versorgungszahlen in der Fläche

Auf einer Fläche von knapp 358.000 km^2^ versorgten die 3469 niedergelassenen Fachärzt*innen für Urologie (FÄ-Uro, in Einzahl FA-Uro) 83.155.031 Einwohner Deutschlands bei einer durchschnittlichen Bevölkerungsdichte von 233 Einwohner/km^2^ [3]. Im Bundesdurchschnitt kam ein FA-Uro auf 98 km^2^ Fläche. Drei- bis 4‑mal höher lag diese Maßzahl in den Flächenländern Mecklenburg-Vorpommern und Brandenburg und deutlich niedriger mit einem Durchschnittswert von 5 km^2^ pro FA-Uro in den Millionenstädten. Im Bundesdurchschnitt versorgte ein FA-Uro 23.577 Einwohner. Höhere Werte fanden sich in Schleswig-Holstein, Mecklenburg-Vorpommern und Brandenburg; niedrigere Werte in Bremen, Berlin und Hamburg. Mit durchschnittlich etwas mehr als 30.000 Einwohner pro FA-Uro im ländlichen Bereich unterschied sich die Versorgungsdichte dort von größeren Städten: In Groß- und Millionenstädten versorgte ein FA-Uro durchschnittlich weniger als 20.000 Einwohner.

### Struktur der urologischen Praxen

**Figure 2:**
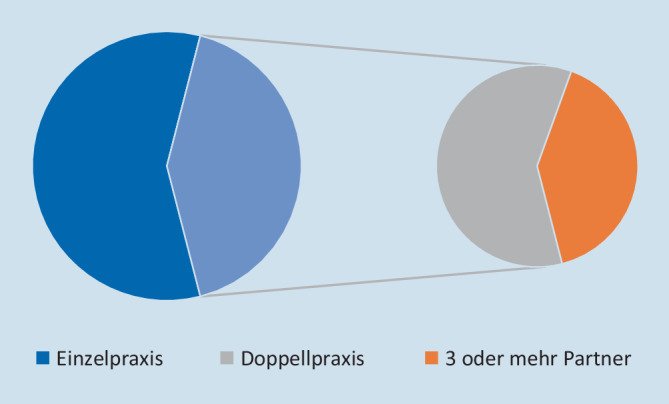

In der Bundesrepublik Deutschland gab es 2077 urologische Praxen. Davon wurden 58 % (1197/2077) als Einzelpraxis und 42 % (880/2077) als Berufsausübungsgemeinschaft (BAG) betrieben. Unter den BAGs waren es in 60 % Doppelpraxen (524/880) und in 40 % (356/880) Praxen mit mindestens 3 Partnern. Abb. 2 zeigt die Verteilung der Praxisstrukturen.

Die größten Zusammenschlüsse waren eine Praxis in Nordrhein-Westfalen mit 10 und eine Praxis in Hessen mit 14 Partnern. Es fanden sich deutliche regionale Unterschiede hinsichtlich der Praxisstruktur und -größe: Während in den eher dünn besiedelten Bundesländern Mecklenburg-Vorpommern, Brandenburg, Sachsen-Anhalt und Saarland ein überdurchschnittlich hoher Anteil an Einzelpraxen bestand (73–95 %), wurden in Bremen und Hamburg nur 40 % der urologischen Praxen als Einzelpraxis geführt.

### Tätigkeit nach Praxisformen

**Figure 3:**
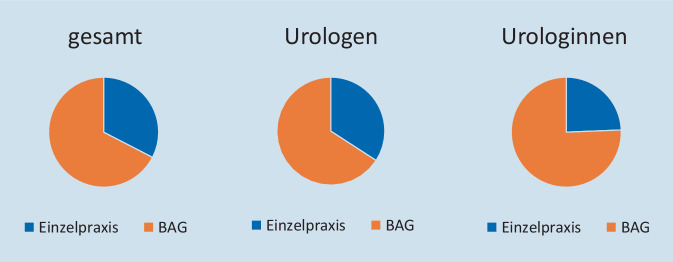

Insgesamt arbeitete die Mehrheit aller FÄ-Uro in BAG (67 %). Lediglich 33 % waren in einer Einzelpraxis tätig. Abb. 3 zeigt zudem die geschlechtsspezifische Tätigkeit je nach Praxisform. 66 % der Urologen in Deutschland waren in BAG und 34 % in Einzelpraxen tätig. Unter den niedergelassenen Urologinnen waren 77 % in einer BAG tätig und 23 % in einer Einzelpraxis. Es zeigte sich hier ein deutlicher Unterschied hinsichtlich der Tätigkeit in Einzelpraxen oder BAG zwischen den Geschlechtern (*p* < 0,001).

Ausnahmen hiervon zeigten sich in Mecklenburg-Vorpommern und Sachsen, wo > 60 % der Urologinnen in Einzelpraxen niedergelassen waren.

### Versorgungsstruktur nach Region

**Figure 4:**
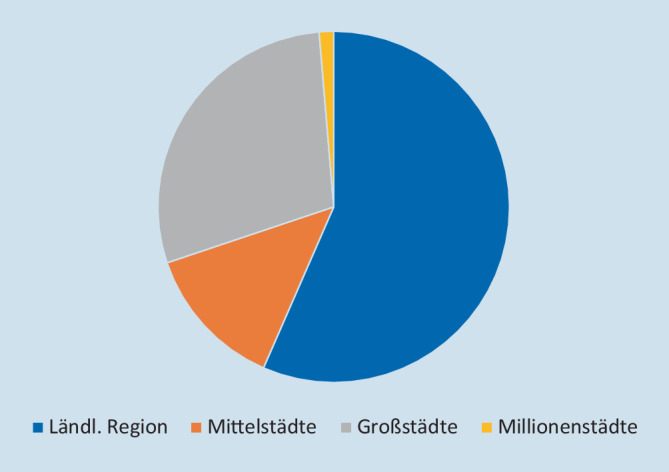

Knapp die Hälfte der Praxisurologie in Deutschland fand im ländlichen Bereich statt, in dem 60 % der deutschen Bevölkerung lebte. Etwas über ein Viertel der FÄ-Uro und der Praxen waren in den Großstädten lokalisiert, in denen 21 % aller Bürger lebten. Weitere 11 % entfielen auf die Mittelstädte und 14 % auf die Millionenstädte mit jeweils 9 % der deutschen Bevölkerung. Abb. 4 zeigt die Verteilung der fachurologischen Praxen nach Standort.

Besonders in Hinblick auf die Praxisstruktur zeigten sich darüber hinaus Unterschiede je nach Lokalisation der Praxen. Bei zunehmender Einwohnerzahl der Region sank der Anteil der in einer Einzelpraxis tätigen FÄ-Uro. Im ländlichen Gebiet waren 40 % der FÄ-Uro in einer Einzelpraxis tätig, in Großstädten 18 % (*p* < 0,001).

**Figure 5:**
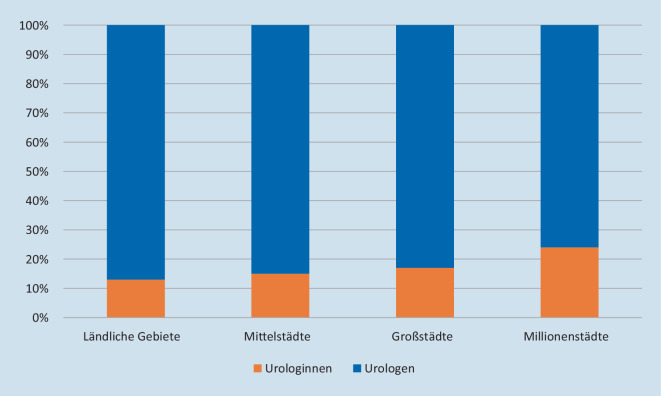

Die Abb. 5 illustriert die Geschlechterverteilung unter allen FÄ-Uro nach Umfeld. Der Anteil der Urologinnen erhöhte sich mit zunehmender Bevölkerungszahl der Standorte von 13 % im ländlichen Bereich über 15 % in den Mittelstädten und 17 % in den Großstädten bis auf 24 % in den Millionenstädten. In Millionenstädten war ein signifikant höherer Anteil an Urologinnen unter allen FÄ-Uro tätig als im ländlichen Bereich (*p* = 0,045).

### Neue und alte Bundesländer

Die neuen Bundesländer nehmen nahezu ein Drittel der Fläche der Bundesrepublik Deutschland ein; dort leben jedoch nur 17 % der Bevölkerung. Die Dichte der urologischen Versorgung war zum Erhebungszeitpunkt in beiden Teilen ähnlich: Ein FA-Uro versorgte durchschnittlich etwa 24.000 Einwohner. Der Anteil der Urologinnen betrug in den alten Bundesländern 14,5 % (461/3004) und in den neuen Bundesländern 21 % (137/635; *p* < 0,001). Auch die Praxisstruktur unterschied sich: Der Anteil der Einzelpraxen lag in den alten Bundesländern bei 31 % und in den neuen Bundesländern bei 48 % (*p* = 0,01).

### Altersstruktur der FÄ-Uro

**Figure 6:**
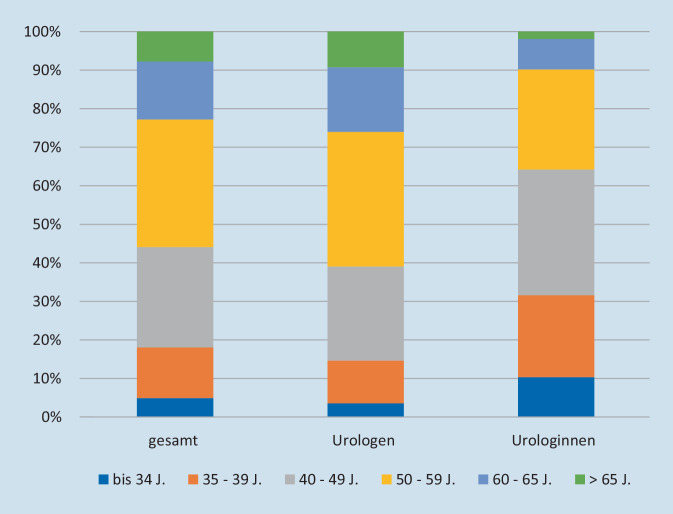

Die Abb. 6 zeigt die Altersstruktur aller Fachärzt*innen für Urologie, die in Deutschland tätig sind. Die hier zugrunde liegenden Daten schließen die in der stationären Versorgung tätigen Kolleg*innen mit ein. Der größte Anteil aller FÄ-Uro (26 % bzw. 33 %) war zwischen 40–49 bzw. 50–59 Jahre alt. Lediglich 5 % waren jünger als 35 Jahre und nur 8 % älter als 65 Jahre. Unter allen männlichen Urologen waren nur 4 % jünger als 35 Jahre; bei allen Urologinnen waren es dagegen 10 % in der jüngsten Altersgruppe (*p* < 0,001).

**Figure 7:**
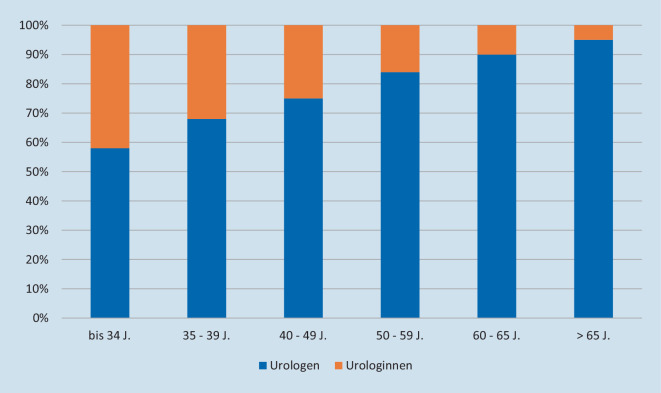

Die Abb. 7 verdeutlicht, dass der Anteil an Urologinnen unter allen FÄ-Uro (ambulant und stationär tätig) umso größer war, je jünger die betrachtete Subgruppe war (*p* < 0,001).

## Diskussion

Aus den Vergleichen der vier Stadtkategorien ergaben sich nennenswerte Unterschiede in dem Anteil der Einzelpraxen sowie der Versorgungszahl zwischen dem ländlichen Bereich und den drei Kategorien der größeren Städte. Im ländlichen Bereich war der Anteil der Einzelpraxen deutlich höher als in den drei städtischen Kategorien, die sich wiederum kaum voneinander unterschieden. Vor allem in den Flächenländern Mecklenburg-Vorpommern, Brandenburg und Sachsen-Anhalt zeigte sich ein besonders hoher Anteil an Einzelpraxen. Analog dazu waren es in den neuen Bundesländern prozentual mehr Fachärzt*innen für Urologie (FÄ-Uro), die in einer Einzelpraxis niedergelassen waren, als im restlichen Bundesgebiet.

In ländlichen Gebieten versorgte ein FA-Uro zudem tendenziell mehr Einwohner als in Städten; wobei hier die Aussagekraft durch unterschiedliche Einzugsgebiete limitiert ist. Patient*innen, die im ländlichen Gebiet wohnen, nehmen auch Versorgungsangebote in städtischen Regionen war.

Darüber hinaus zeigten sich in der geschlechtsspezifischen Betrachtung deutliche Unterschiede zwischen männlichen Fachärzten für Urologie und den weiblichen Kolleginnen.

Fachärztinnen für Urologie arbeiteten zum Erhebungszeitpunkt demnach häufiger in Kliniken. Unter den Urologinnen arbeiteten diejenigen, die in niedergelassenen Bereichen tätig sind, häufiger als ihre männlichen Kollegen in Berufsausübungsgemeinschaften (BAG). Zudem fand sich in Millionenstädten und in den neuen Bundesländern ein signifikant höherer Anteil an Urologinnen. In der Analyse der Altersstruktur aller FÄ-Uro (niedergelassene und stationär tätige Kolleg*innen) zeigte sich eine Verschiebung der Geschlechterverteilung: Je jünger die betrachtete Alterssubgruppe war, desto höher war der Anteil an Urologinnen unter allen FÄ-Uro.

Unsere Arbeit belegte mehrere Trends, die auch aus anderen medizinischen Fachrichtungen bekannt sind: Zum einen zeigte der im ländlichen Bereich deutlich höhere Anteil an Einzelpraxen die besondere Funktion der Einzelpraxis im Versorgungssystem. Einzelpraxen gewährleisteten die urologische Versorgung in den dünner besiedelten Regionen Deutschlands. Durch sie wurde die dezentrale, flächendeckende Versorgung mit tolerablen Anfahrtswegen für die Patient*innen sichergestellt. Ein FA-Uro konnte so ein größeres Einzugsgebiet von Patient*innen versorgen. In großen Städten dagegen war es möglich, durch Kooperationen mehrerer Partner betriebswirtschaftliche Vorteile einer BAG zu nutzen und das diagnostische und therapeutische Spektrum wirtschaftlich rentabel auszuweiten.

Darüber hinaus waren v. a. die geschlechtsspezifischen Unterschiede beachtenswert: Im Durchschnitt arbeiteten die Fachärztinnen für Urologie vermehrt im stationären Umfeld bzw. in BAG und in städtischen Gebieten. Dieses Ergebnis deckte sich mit Erkenntnissen zu Urologinnen in den USA [8, 9].

Auch eine aktuell veröffentlichte Umfrage von Himmler et al. [10] zeigte einen geringeren Anteil an Frauen unter den niedergelassenen FÄ-Uro und bestätigt damit die hier vorliegenden Daten.

Eine mögliche Erklärung könnte die weiterhin bestehende soziokulturelle Prägung der Geschlechter sein. Nach wie vor sind es eher die weiblichen Kolleginnen, die beispielsweise Elternzeit in Anspruch nehmen, längere Berufspausen einlegen und bei Berufstätigkeit häufiger in Teilzeit arbeiten [11]. All dies ist weniger gut mit der selbstständigen Tätigkeit in einer Einzelpraxis vereinbar.

Als alleinige Erklärung scheint diese Hypothese jedoch zu kurz zu greifen, weil sich sehr wahrscheinlich Gendereffekte und Generationsunterschiede überlagern. Innerhalb der jüngeren Subgruppen lag der Anteil der Urologinnen unter allen FÄ-Uro deutlich höher, sodass beim Vergleich der beiden Geschlechter stets eine Altersbias resultierte.

Der im Rahmen der hier vorliegenden Studie beobachtete Unterschied zwischen männlichen und weiblichen Kolleg*innen lässt sich ebenso als Unterschied zwischen jüngeren und älteren Kolleg*innen verstehen. Pace et al. [12] konnten zeigen, dass sich über das traditionell stereotypische Rollenverständnis hinaus das Selbstverständnis der männlichen Teile der jüngeren Generation geändert hat [11]: Mehr Zeit für Privates und die Familie ist ebenso Priorität wie der Wunsch in großen Städten und in größeren Teams zu arbeiten [9–11].

Unter anderem deswegen könnten beispielsweise Arbeitsverhältnisse in Kliniken oder als angestellte FÄ-Uro in Niederlassung für jüngere FÄ-Uro attraktiver sein, als sie das für vorherige Generationen waren. Bei insgesamt eher geringem Anteil an Urologinnen fällt die alters- bzw. generationenbedingte Verschiebung der Präferenzen bei geschlechtsspezifischer Betrachtung der Daten deutlicher ins Gewicht. Unter Umständen zeigt sich in einigen Jahrzehnten ein ausgeglicheneres Bild, wenn sich der Anteil der Urologinnen über alle Altersgruppen gleichmäßiger verteilt haben wird. Im Zuge der zunehmenden Anzahl an Frauen unter den FÄ-Uro könnte in Zukunft der Unterschied zwischen verschiedenen Generationen bedeutsamer erscheinen als der Unterschied zwischen den Geschlechtern [9].

Um die Zukunft der Urologie im Allgemeinen und der niedergelassenen Urologie im Besonderen positiv zu gestalten, sollten wir den sich ändernden Wünschen Rechnung tragen. Flexible Arbeitszeiten, die eine gute Work-Life-Balance ermöglichen, sowie leicht verfügbare Kinderbetreuung spielen hierbei sicherlich gesamtgesellschaftlich große Rollen. Aber auch Konzepte eines geteilten Kassensitzes und geregelte Vertretungsmöglichkeiten bei Abwesenheit können nicht-monetäre Anreize sein, den Schritt in die Niederlassung attraktiver zu gestalten. Wie sich aus dem zugehörigen Online-Supplement ergibt, tragen die zeitgeschichtlichen Entwicklungen diesen Wünschen Rechnung: Zwischen 1980 und 2020 hat sich der Anteil der in Einzelpraxen Tätigen halbiert, während sich der Anteil der Urolog*innen in Berufsausübungsgemeinschaften nahezu verachtfacht hat. Die dynamischsten Trends der vergangen 20 Jahre waren die Praxistätigkeit von Urologinnen, die Tätigkeit von Urolog*innen in BAG und die Zunahme der Angestelltentätigkeit von Urolog*innen. In welchem Umfang sich das Prinzip der Einzelpraxis gerade im ländlichen Raum in Zukunft wird fortführen lässt, bleibt dabei abzuwarten. Mit berufspolitischem Engagement – wie beispielsweise dem verstärkten Fokus auf Chancengleichheit über die AG Urologinnen der DGU – kann die Urologie auch in Zukunft ein attraktives Berufsfeld bleiben.

## Stärken und Limitationen

Leider war die Altersstruktur der niedergelassenen Kolleg*innen nicht separat erhebbar, sodass wir hierzu nur Daten zu allen Urolog*innen berichten konnten. Aufgrund des deskriptiven Charakters sind prinzipiell keine ursächlichen Erklärungen möglich. Dennoch schafft die vorliegende Arbeit erstmalig eine umfassende Beschreibung der ambulanten Versorgungsstruktur in der deutschen Urologie. Insbesondere für zukünftige vergleichende Analysen kann sie als wertvolle Grundlage dienen. Hierfür sind die Rohdaten auch als *Online-Supplement* verfügbar. Dieses enthält zusätzlich weitere Aspekte der ambulanten Urologie (Mehrfachtätigkeit, MVZ, Angestelltentätigkeit, Privatärzte, Muster der urologischen Versorgung, zeitgeschichtliche Entwicklung und Trends) und eine Analyse der Belegarzttätigkeit.

## Bedeutungen für die Praxis

Die jüngere Generation an FÄ-Uro hat offensichtlich andere Wünsche an die Art zu arbeiten als die Kolleg*innen zuvor. Zugunsten von wünschenswerten Rahmenbedingungen scheinen sie eher bereit zu sein, auf einen Teil des möglichen Einkommens zu verzichten. Einzelpraxen sind gerade in weniger besiedelten Regionen ein wichtiges Mittel zur Gewährleistung der flächendeckenden Versorgung, dürften aber aufgrund der Gender- und Generationseffekte weniger beliebt werden.

Wie sich dies auf die zukünftige Struktur der urologischen Versorgung auswirken wird, bleibt abzuwarten. Im besten Fall lässt sich die Entwicklung berufspolitisch positiv gestalten.

## Fazit für die Praxis

- Die Mehrheit der aktuell tätigen Fachärzte für Urologie ist in der Niederlassung tätig.
- In der Bundesrepublik Deutschland gibt es zurzeit etwa 2100 urologische Praxen, die die flächendeckende Versorgung aller Einwohner sicherstellen.
- Das Muster der fachurologischen Versorgung unterscheidet sich dabei je nach Einwohnerzahl der betrachteten Region und Bundesland.
- Zudem zeigen sich Trends, die die zukünftige Arbeit in urologischen Praxen beeinflussen werden. Hierbei sind Gendereffekte genauso zu beachten wie Generationenunterschiede.

## Supplementary Information




