# Supplementary material for: Structure of outpatient urology care in Germany
Source: Urologie. 2023 Mar 9;62(5):503–9. [Article in German] doi: 10.1007/s00120-023-02048-x (PMC10160173; doi:10.1007/s00120-023-02048-x)
Supplement: Supplementary file 1 [file 120_2023_2048_MOESM1_ESM.docx]

Online Supplement

zur Publikation

**Versorgungsstruktur der ambulanten Urologie in Deutschland**

Helmut Haas^1*^, Laura Müller^2*^, Thomas Speck^3^, Maurice-Stephan Michel^4^, Johannes Huber^2^

Ergänzend zu den Analysen der Hauptarbeit werden hier die zugrunde liegenden Datenwerken präsentiert und weitere Aspekte der Praxisurologie bearbeitet, einschließlich des zeitgeschichtlichen Kontexts und den daraus abgeleiteten Trends. Abschließend erfolgt eine Analyse des Belegarztwesens.

Praxisurologie

Die Gesamtdaten zur Praxisurologie in Deutschland finden sich in Tab. 1.

Zusätzliche Einzelaspekte werden im Folgenden behandelt.

**Tätigkeit von Fachärzten für Urologie in mehr als einer Praxis.** 178 FÄ-Uro und damit etwa jeder zwanzigste sind an mehr als einem Standort tätig, 90% von ihnen an einem weiteren Standort, 9% an zwei weiteren Standorten und 1% an drei weiteren Standorten. Das Spektrum reicht vom Betrieb einer Zweigpraxis bis zur Tätigkeit von FÄ-Uro in mehreren Großpraxen. Die Tätigkeit eines FA-Uro in mehreren Praxen ist mit 7% anteilig am häufigsten im ländlichen Bereich, sinkt über 5% in Mittelstädten und 3% in Großstädten ab auf einen Anteil von unter 1% in Millionenstädten.

**Medizinische Versorgungszentren (MVZ).** Es wurden 285 FÄ-Uro identifiziert, die in 152 MVZs tätig waren mit der Besetzung von 1 – 7 FÄ-Uro im MVZ. Etwa ein Drittel der FÄ-Uro waren als einzige Urologen zu erkennen in einem damit wohl fachübergreifenden MVZ. 10 MVZs waren aufgrund der Benennung oder der Personen der Betreiber als ausschließlich urologisch zu identifizieren.

Häufig finden sich Medizinische Versorgungszentren in Thüringen und Sachsen, wo jeder dritte bzw. fünfte FA-Uro in einem MVZ arbeitet. Vielgestaltig ist die Zusammensetzung der Betreiber, soweit dies aus dem Namen des MVZ erkennbar war. In 6 Fällen waren dies FÄ-Uro, in 19 Fällen Kliniken, darüber hinaus kommunale, karitative und kirchliche Einrichtungen. Häufig sind es neben den großen Kliniksbetreibern wie Helios und Asklepios öffentlich weitgehend unbekannte Betreiber aus dem offensichtlich privatwirtschaftlichen Bereich, die regional, aber auch bundesweit agieren. Soweit aus dem Namen erkennbar, sind sie als GbR, GmbH, gGmbH oder als Stiftung tätig sind. Eine eindeutige AG war nicht zu erkennen.

Tab. 1: Grundstrukturen der Praxisurologie in Deutschland

**Angestelltentätigkeit.** Der Bericht der KBV zum 31.12.2020 [3] weist 639 FÄ-Uro als Angestellte aus, davon 192 Urologinnen (30%) und 447 Urologen. Der Anteil der Urologinnen ist somit höher als nach der Geschlechterverteilung aller FÄ-Uro zu erwarten.

Die Zahl der Angestellten verteilt sich etwa hälftig auf die Anstellung in MVZs und Praxen. Urologen sind deutlich häufiger in MVZs angestellt als ihre weiblichen Kollegen, die offensichtlich die Anstellung in Praxen bevorzugen.

**Ermächtigungen.** Kliniksärzte können von den Kassenärztlichen Vereinigungen ermächtigt werden, in begrenztem Umfang und beschränkt auf spezielle Leistungen gesetzlich versicherte Patienten zu behandeln. Nach den KBV-Angeben zum 31.12.2020 [3] gab es 285 ermächtigte Kliniksurologen, davon 51 Frauen. Verglichen mit ihrem Anteil an ihrer Tätigkeit im klinischen Sektor sind damit Urologinnen im geringeren Umfang ermächtigte Ärzte als ihre männlichen Kollegen.

**Privatärzte.** Privatärzte sind selbständige niedergelassene Fachärzte für Urologie ohne vertragsärztliche Zulassung, die ihre Leistungen dem Patienten direkt in Rechnung stellen. Es wurden in Deutschland 199 Privatärzte identifiziert. Ihre Zahl liegt bei 5,8%, verglichen mit der Zahl der Kassenärzte. Es findet sich eine Häufung in Hessen und Hamburg (jeweils 10%) und Bayern (9%). Ein hoher Anteil findet sich im Großraum Frankfurt/Main (39%) und München (23%), während in den übrigen Millionenstädten und den Landeshauptstädten ihr Verhältnis nicht über 10% der Kassenärzte hinausgeht

**Neue und alte Bundesländer** (Abb. 2). Die neuen Bundesländer nehmen nahezu ein Drittel der Fläche der Bundesrepublik Deutschland ein, dort lebt jedoch nur 17% ihrer Bevölkerung. Im Vergleich zu 274 Einwohner/km^2^ in den alten ist die Bevölkerungsdichte in den neuen Ländern mit 129 Einwohner/km^2^ deutlich geringer. Die urologische Versorgung ist in beiden Bereichen ähnlich, in beiden versorgt ein FA-Uro um 24.000 Einwohner. 83% aller FÄ-Uro arbeiten in 79% aller deutschen Praxen in den alten Bundesländern, in den neuen Bundesländern 17% von allen in 21% aller Praxen.

Vier Fünftel der Praxisurologie in Deutschland findet, gemessen an der Zahl der FÄ-Uro, den Anteilen der Praxen und der Arbeitsplätze in den alten Bundesländern statt. Häufiger sind die FÄ-Uro dort in BAGs tätig. Über zwei Drittel der Praxen in den neuen Ländern sind Einzelpraxen im Vergleich zu etwa der Hälfte in den alten Bundesländern. Entsprechend arbeitet jeder dritte FA-Uro in den alten Ländern in einer Einzelpraxis, in den neuen Ländern jeder zweite. Hingegen sind Groß-BAGs in den neuen Ländern selten.

Der Anteil der Urologinnen ist in den neuen Ländern deutlich höher, und sie sind häufiger in Einzelpraxen tätig als in den alten.

Tab. 2: Alte vs. neue Bundesländer und Muster der urologischen Versorgung nach Stadtkategorien

**Muster der urologischen Versorgung** (Tab. 2)**.** Aus den Daten der vier Stadt-kategorien ergeben sich nennenswerte Unterschiede in dem Anteil der Einzelpraxen und der Zahl der Ärzte/Praxis zwischen dem ländlichen Bereich einerseits und den 3 Kategorien der größeren Städte andererseits. Im ländlichen Bereich ist der Anteil der Anteil der Einzelpraxen (63% vs. 52%) höher und die Zahl der Ärzte/Praxis (1,5 vs. 1,9) niedriger als in den drei anderen Kategorien, die sich wiederum kaum voneinander unterscheiden.

Aus dieser Betrachtung ergeben sich zwei Grundmuster der ambulanten urologischen Versorgung:

1. Muster **„Dezentrale Flächendeckung“:** empirisch charakterisiert durch einen hohen Anteil von Einzelpraxen und eine geringere Zahl von FÄ-Uro/Praxis in Regionen mit niedriger Bevölkerungsdichte

2. Muster **„Urbane Konzentration“:** empirisch charakterisiert durch einen niedrigen Anteil der Einzelpraxen, jedoch einer höheren Zahl von FÄ-Uro/ Praxis bei hoher Bevölkerungsdichte.

Diese empirischen Befunde belegen die besondere Funktion der Einzelpraxis im Versorgungssystem, die urologische Versorgung in den dünner besiedelten Regionen Deutschlands zu gewährleisten. Durch sie wird mit geringstem personellem und organisatorischem Aufwand die flächendeckende Versorgung mit tolerablen Anfahrtswegen für den Patienten sichergestellt und gleichzeitig den arbeitenden FÄ-Uro ein Auskommen gesichert.

In großen Städten andererseits mit vergleichsweisen kurzen Wegen ist es möglich, durch Kooperationen mehrerer Partner betriebswirtschaftliche Vorteile zu nutzen und das diagnostische (z.B. Fusionsbiopsie, Urodynamik) und therapeutische Spektrum (größere ambulante Operationen, onkologische Schwerpunktbildung) wirtschaftlich rentabel auszuweiten.

Zeitgeschichtlicher Kontext

Die Anzahl und die Form der Tätigkeit der FÄ-Uro verändern sich zwischen 1980 und 2020 deutlich (Tab. 3)

Das Zahlenwerk der Kassenärztlicher Bundesvereinigung (KVB) [1,2] fokussiert die zugelassenen FÄ-Uro. Ihre Zahl stieg zwischen 1980 und 2020 auf das 2,5fache. 1980 waren 93% von ihnen in einer Einzelpraxis tätig, und BAGs waren mit maximal 3% aller Praxen die Ausnahme. Seit 2000 sinkt die Zahl der in Einzelpraxis tätigen FÄ-Uro kontinuierlich ab. 2015 überschritt die Zahl der FÄ-Uro in einer BAG erstmals die in einer Einzelpraxis. Die Zahl der zugelassenen Urologinnen, 1980 mit 6 Urologinnen noch eine Ausnahme, stieg in diesen 40 Jahren sehr deutlich auf 507 an.


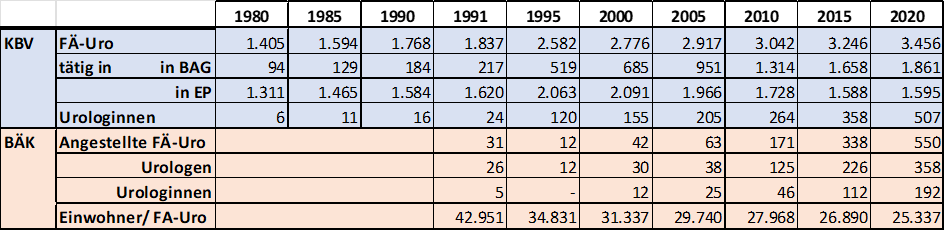


Tab. 3: Tätigkeitsstrukturen in der Urologischen Praxis zwischen 1980 und 2020

Die Datenbank der Bundesärztekammer (BÄK) [3] ab 1991 lässt die Zahlenent-wicklung von angestellten FÄ-Uro beurteilen. Die Zahl der angestellten FÄ-Uro stieg von 31 auf 550 im Jahr 2020, die der Urologinnen stärker als die der Urologen. Die Zahl der Einwohner, die ein ambulant tätiger FA-Uro versorgt, sank von bundesweit 42.951 im Jahr 1991 auf 25.337 im Jahr 2020. Der Ausgangswert in Verbindung mit einem hohen Anteil der in der Einzelpraxis Tätigen (88%) lag damit noch deutlich höher als der, den wir heute vom ländlichen Bereich kennen, in dem die Einzelpraxis hohe Anteile einnimmt. Die ist ein weiterer Beleg für die Funktion der Einzelpraxis als Mittel zur flächendeckenden urologischen Versorgung.

Tab. 4: Bemerkenswerte Trends in der praxisurologischen Tätigkeit

**Trends** (Tab. 4)**.** Um mögliche Trends zur Zukunftseinschätzung zu erkennen, wurden der Verlauf zwischen dem Jahr 2000 als Ausgangs- und dem Jahr 2020 als Endpunkt analysiert.

In diesem Zeitraum findet sich nur eine geringe Zunahme der Gesamtzahl der zugelassenen FÄ-Uro, ebenso die der Urologen und ihr Anteil an der Niederlassung mit einer Steigerungsrate von 0% - 1% pro Jahr. Der Anteil der Einzelpraxis sank jeweils um 1%/Jahr. Dagegen hat die Tätigkeit in einer BAG mit einer jährlichen Steigerungsrate von 9% deutlich zugenommen.

Merklich gesteigert haben sich die Zahl der Urologinnen, deren Tätigkeit als Kassenärztinnen, aber auch als Angestellte mit jährlichen Steigerungsraten zwischen 6% und 11%. Die Angestelltentätigkeit hat sowohl bei den Urologinnen wie den Urologen dynamisch zugenommen mit jährlichen Steigerungsraten von 55% bzw. 75%. 2020 waren 12% aller Urologen und 35% aller Urologinnen im Angestelltenverhältnis tätig.

Dynamische Entwicklungen sehen wir also bei den Urologinnen, der Tätigkeit in einer BAG und der Angestelltentätigkeit, während die Zahlen für die Urologen und die Tätigkeit als Kassenarzt nur geringfügig steigen und die Einzelpraxis offensichtlich an Attraktivität verliert.

Belegärztliche Versorgung

**Belegärztliche Tätigkeit (**Tab. 5)**.** Belegärzte sind niedergelassene Vertragsärzte, die im ambulanten wie auch im stationären Sektor selbständig tätig sind [4]. Bundesweit fanden sich 415 Ärzte/Ärztinnen in Belegtätigkeit, davon 27 Belegärztinnen (7%), die insgesamt in 162 Kliniken tätig sind. Der Anteil der Belegärzte an allen FÄ-Uro beträgt 12%. Der Anteil der Urologinnen unter den Belegärzten ist mit 7% niedriger als unter allen FÄ-Uro. Im Bundesdurchschnitt kommen 2,6 Belegärzte auf eine Klinik. Am häufigsten ist die Konstellation von 2 Belegärzten/Klinik. Nur in jeder zehnten Klinik sind 5 und mehr Belegärzte tätig. Die höchste Zahl von Ärzten in einer Klinik findet sich mit je 13 Belegärzten in einer Klinik in Bayern und einer in Baden-Württemberg.

78% aller Belegkliniken sind in Städten mit unter 50.000 Einwohnern lokalisiert, davon über die Hälfte in Gemeinden mit unter 20.000 Einwohnern. Dies belegt die besondere Bedeutung der Belegkliniken in der wohnortnahen stationären Patientenversorgung gerade im ländlichen Bereich. Hier dominieren mit einem Anteil von 71% die Abteilungen mit 1 – 2 Belegärzten, deren Anteil in Großstädten auf 43% absinkt.

Tab. 5: Belegärztliche Tätigkeit in der Bundesrepublik Deutschland, im Vergleich der alten und neuen Bundesländer und abhängig von den Stadtkategorien

Literatur:

1. Statistische Informationen aus dem Bundesarztregister. Bundesgebiet insgesamt. Stand: 31.12.2020. Kassenärztliche Bundesvereinigung, Berlin. [www.kbv.de/media/sp/2020-12-31_BAR_Statistik.pdf](http://www.kbv.de/media/sp/2020-12-31_BAR_Statistik.pdf)

2. An der vertragsärztlichen Versorgung teilnehmende Urologen. aus Bundesarztregister der KBV. [www.kbv.de/html/bundesarztregister.php](http://www.kbv.de/html/bundesarztregister.php)

3. Ärztestatistik der Vorjahre der Bundesärztekammer [https://www.bundesaerztekammer.de/ueber-uns/aerztestatistik/aerztestatistik-der-vorjahre/](https://www.bundesaerztekammer.de/ueber-uns/aerztestatistik/aerztestatistik-der-vorjahre/%20%20%20%20%20%20)

4. Berufspolitik BvDU. Urologie 61, 1157–1167 (2022). https://doi.org/10.1007/s00120-022-01946-w
